# Supplementary material for: Discovery of a New Microbial Origin Cold-Active Neopullulanase Capable for Effective Conversion of Pullulan to Panose
Source: Int J Mol Sci. 2022 Jun 22;23(13):6928. doi: 10.3390/ijms23136928 (PMC9267027; doi:10.3390/ijms23136928)
Supplement: Supplementary file 1 [file ijms-23-06928-s001.zip › ijms-1762780-supplementary.pdf]

Supplementary Material

Discovery a new microbial origin cold-active neopullulanase capable for effective conversion pullulan to panose

Meixing Wang[1,2,†], Huizhen Hu[2,3,†], Buyu Zhang[2], Yang Zheng[2], Pan Wu[2], Zhenghui Lu[2] and Guimin Zhang[1,*]

[1]College of Life Science and Technology, Beijing University of Chemical Technology, Beijing 100029, China

[2]State Key Laboratory of Biocatalysis and Enzyme Engineering, School of Life Sciences, Hubei University, Wuhan, Hubei 430062, China

[3]Yunnan Province Engineering Research Center for Functional Flower Resources and Industrialization, College of Landscape Architecture and Horticulture Sciences, Southwest Forestry University, Kunming 650224, China

*Correspondence: zhangguimin@buct.edu.cn, zhangguimin6@hotmail.com; College of Life Science and Technology, Beijing University of Chemical Technology, Beijing 100029, China

[†]These authors contributed equally to this study.

Table S1 HPLC-ELSD analysis of hydrolysis products from α-cyclodextrin, β-cyclodextrin and γ-cyclodextrin by Amy117.

Types	Substrates	Time (min)	Characteristic peak area	Maltose/Glucose
Samples	α-cyclodextrin	12.668	1517.200	2.584
		23.542	3919.800	
	β-cyclodextrin	12.690	1561.100	2.680
		23.549	4184.400	
	γ-cyclodextrin	12.603	1457.300	2.954
		23.335	4305.500	
Standards	Glucose	12.641	1531.500	/
	Maltose	22.729	3867.100	/
	Maltotriose	37.723	2551.700	/

Table S2 Information of some reported enzymes that show high sequence homology with Amy117.

Name and GenBank number	Source	Mol. Wt. (kDa)	Opt. Temp (ºC)	Opt. pH	Reaction and products			Reference	Identity (compare with Amy117)
					Pullulan	Soluble starch	Cyclodextrin		
MAase (ACN79585.1)	*Parageobacillus caldoxylosiyticus* TK4	70	50	7.0	-	-	Maltose, glucose and other maltooligosaccarides	Kolcuogl et al. 2010	64%
MAase (AFM43699.1)	*Geobacillus thermoleovorans*	72.5	80	5.0-9.0	ND	Mainly maltose	Mainly maltose and glucose	Mehta et al. 2013	63%
MAase BSMA (AAC46346.1)	*Geobacillus stearothermophilus* ET1	70	55	6.0	Glucose, maltose, panose	Mainly maltose	Mainly maltose	CHA et al. 1998	63%
MAase ThMA (AAC15072.1); (PDB code: 1SMA)	*Thermus sp. strain IM6501*	68	60	6.0	Glucose, maltose, panose	maltose and glucose	maltose and glucose	Kim et al. 1999	62%
NPase bsNpl (AAK15003.1)	*Geobacillus stearothermophilus* IMA6503	65	55	6.0	Glucose, maltose, panose	Mainly maltose	Glucose, maltose	Cheong et al. 2002	61%
CDase AfCda13 Amy98 (AAX29991.1)	*Anoxybacillus flavithermus*	73	55-60	6.0	Panose	Mainly Glucose and maltose	Mainly maltose and Some glucose	Turner et al. 2005	63%

CDase Amy132 (ACA48225.1)	Environmental DNA	69	50-65	-	Panose	Glucose, maltose, maltotriose	Glucose, maltose, maltotriose	Labes et al. 2008	61%

Table S3 Information of all other reported enzymes that producing panose as the only product from pullulan.

Name and GenBank number	Source	Mol. Wt. (kDa)	Opt. Temp (ºC)	Opt. pH	Reaction and products			Reference	Identity (compare with Amy117)
					Pullulan	Starch	Cyclodextrin		
CDase AfCda13 Amy98 (AAX29991.1)	*Anoxybacillus flavithermus*	73	55-60	6.0	Panose	Mainly Glucose and Maltose	Mainly maltose and Some glucose	Turner et al. 2005	63%
CDase Amy132 (ACA48225.1)	Environmental DNA	69	50-65	-	Panose	Glucose, maltose, maltotriose	Glucose, maltose, maltotriose	Labes et al. 2008	61%
NPase (AJT51278.1)	*Lactobacillus mucosae* LM1	70	37	6.0	Panose	-	-	Marilen et al. 2016	45%
α-amylase TVAII (BAA02473.1); (PDB code: 1JI2)	*Thermoactinomyces vulgaris* R-47	64		5.5-6.0	Panose	Maltose	Maltose	Mizuno et al. 2004	42%
NPase CdaA (CAB40078.1)	*Alicyclobacillus acidocaldarius* ATCC27009	66	55	5.5	Panose	-	-	Matzke et al. 2000	42%
CDase LsCda13 Amy92 (AAX29990.1)	*Laceyella sacchari*	69	55	6.0	Panose	Mainly Glucose and maltose	Mainly maltose and some glucose	Turner et al. 2005	41%
NPase Amy29 (ACA48224.1)	Environmental DNA	55	50-65	-	Panose	ND	ND	Labes et al. 2008	40%

NPase (AAD05199.1)	*Paenibacillus polymyxa* CECT 155	58	50	6.0	Panose	-	-	Yebra et al. 1999	26%
α-amylase Rbamy5 (SPE91476.1)	*Ruminococcus bromii* ATCC 27255	60	45	6.0	Panose	Glucose, maltose	Glucose, maltose	Jung et al. 2019	21%
MAase CoMA (AVC05420.1)	*Corallococcus* sp. EGB	60	50	7.0	Panose	Maltose	Maltose	Zhou et al. 2018	18%

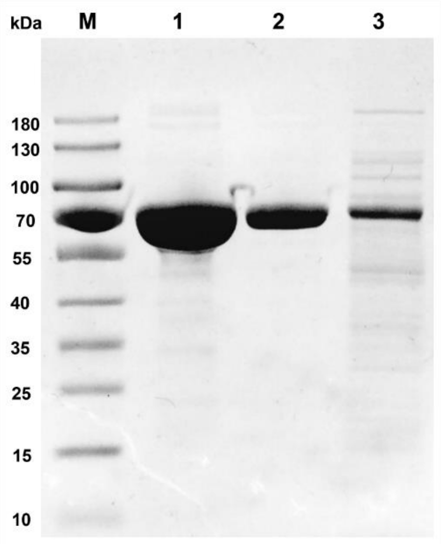

Figure S1. SDS-PAGE analysis of the recombinant Amy117 at each purification step. Samples were resolved on 12% polyacrylamide gel and then stained with Coomassie Blue R-250. Lane 1, purifi-cation Amy117 after desalting; lane 2, purified Amy117 after Ni-affinity column chromatography; lane 3, cellular proteins from the crude extract and Lane M, protein molecular weight markers.

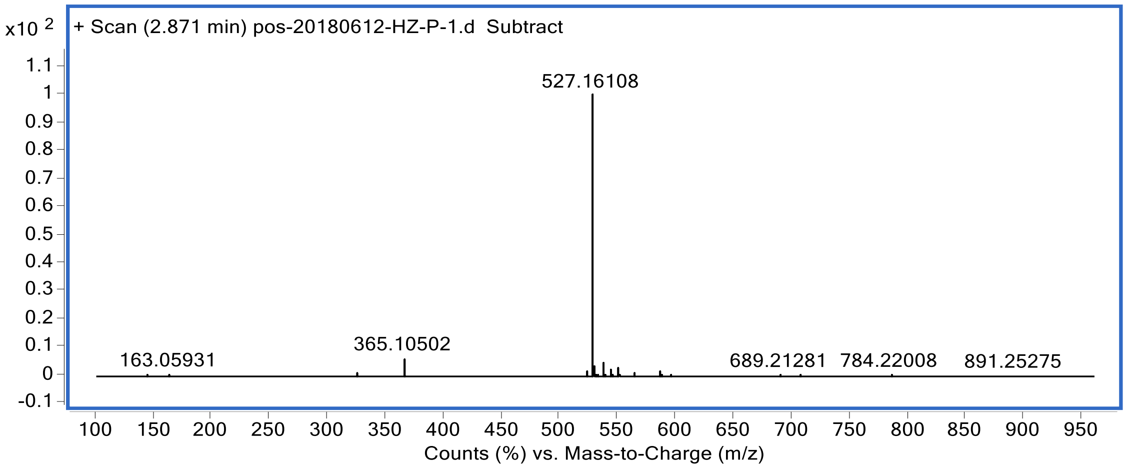

Figure S2. LC-MS on the hydrolytic product from pullulan of Amy117.

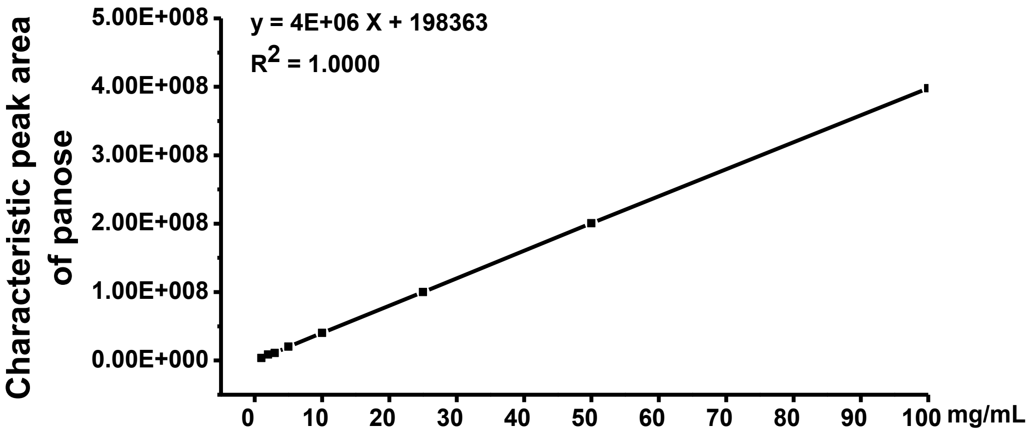

Figure S3. The standard curve of the concentration of panose (the abscissa values) vs the characteristic peak areas (ordinate values).

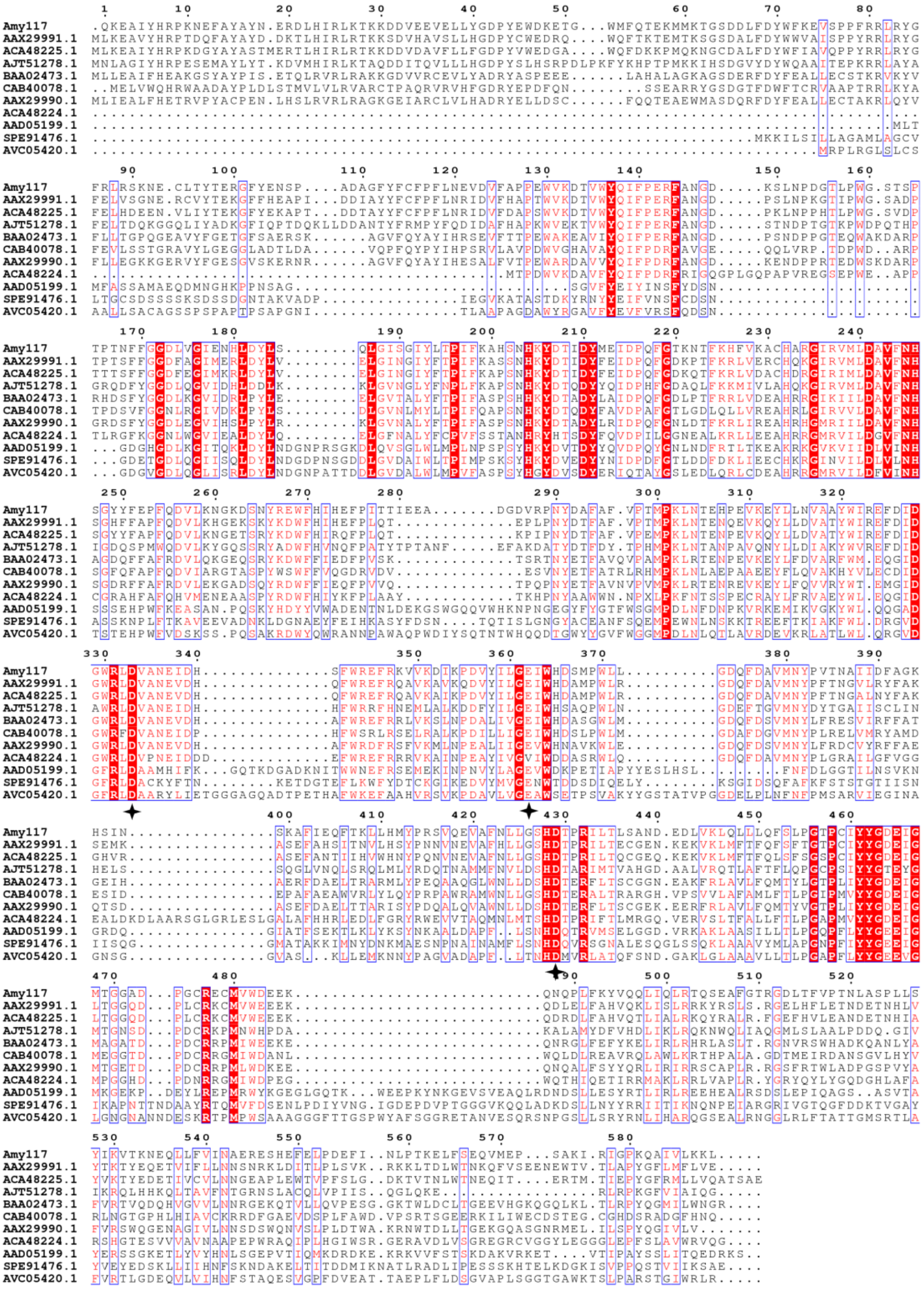

Figure S4. Sequence alignment of Amy17 from *Bacillus pseudofirmus 703* with other neopullulanases that producing panose as the only product from pullulan. Numbers on the left are the positions of the first amino acids in each line. The listed sequences include the NPase Amy98 from *A. flavithermus* (AAX29991.1, 63%), MAase from a *Thermus* strain (ThMA), NPase Amy132 from environmental DNA (ACA48225.1, 61%), NPase from *Lactobacillus mucosae* LM1 (AJT51278.1, 45%), α-amylase TVAII from *Thermoactinomyces vulgaris* R-47 (PDB: 1WZM_A, 42%), NPase CdaA from *Alicyclobacillus acidocaldarius* ATCC27009 (CAB40078.1, 42%), CDase LsCda13 Amy92 from *Laceyella sacchari* (AAX29990.1, 41%), NPase Amy29 from environmental DNA (ACA48224.1, 40%), NPase from *Paenibacillus polymyxa* CECT 155 (AAD05199.1, 26%), α-amylase Rbamy5 from *Ruminococcus bromii* ATCC 27255 (SPE91476.1, 21%), MAase CoMA from *Corallococcus* sp. EGB (AVC05420.1, 18%). The catalytic triads (DED) are marked with the diamonds.

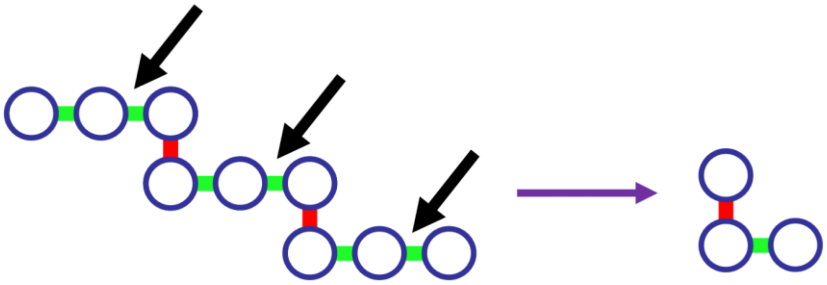

Figure S5. The reaction from pullulan to panose. Amy117 cleave α-1, 4-linkages of pullulan to produce panose. Green bond is the α-1, 4 glycosidic bond, red bond is the α-1, 6 glycosidic bond.
